# Thermal and Mechanical Behavior of Hybrid Polymer Nanocomposite Reinforced with Graphene Nanoplatelets

**DOI:** 10.3390/ma8085262

**Published:** 2015-08-24

**Authors:** Minh-Tai Le, Shyh-Chour Huang

**Affiliations:** 1 Faculty of Mechanical Engineering, Ho Chi Minh City University of Technology and Education, No.1, Vo Van Ngan, Thu Duc District, Ho Chi Minh City 70000, Vietnam; E-Mail: tailm@hcmute.edu.vn; 2 Department of Mechanical Engineering, National Kaohsiung University of Applied Sciences, 415 Chien Kung Road, Sanmin District, Kaohsiung 80778, Taiwan

**Keywords:** nanocomposite, graphene nanoplatelets, epoxy, unsaturated polyester, tensile strength, thermal conductivity, morphology

## Abstract

In the present investigation, we successfully fabricate a hybrid polymer nanocomposite containing epoxy/polyester blend resin and graphene nanoplatelets (GNPs) by a novel technique. A high intensity ultrasonicator is used to obtain a homogeneous mixture of epoxy/polyester resin and graphene nanoplatelets. This mixture is then mixed with a hardener using a high-speed mechanical stirrer. The trapped air and reaction volatiles are removed from the mixture using high vacuum. The hot press casting method is used to make the nanocomposite specimens. Tensile tests, dynamic mechanical analysis (DMA) and thermogravimetric analysis (TGA) are performed on neat, 0.2 wt %, 0.5 wt %, 1 wt %, 1.5 wt % and 2 wt % GNP-reinforced epoxy/polyester blend resin to investigate the reinforcement effect on the thermal and mechanical properties of the nanocomposites. The results of this research indicate that the tensile strength of the novel nanocomposite material increases to 86.8% with the addition of a ratio of graphene nanoplatelets as low as 0.2 wt %. DMA results indicate that the 1 wt % GNP-reinforced epoxy/polyester nanocomposite possesses the highest storage modulus and glass transition temperature (T_g_), as compared to neat epoxy/polyester or the other nanocomposite specimens. In addition, TGA results verify thethermal stability of the experimental specimens, regardless of the weight percentage of GNPs.

## 1. Introduction

Graphene nanoplatelets (GNPs) are a type of filler in the new generation of reinforced materials. Producing high-purity and large amounts of GNPs has a highcost, and because of the issues related to their stability during the process of fabricating nanocomposite materials, researchers have recommended using them in a pristine state, *i.e.,* not to be handled as a functional group [1].

Relating to the matrix for the construction of the composite, thermoset epoxy is the top choice because of its excellent thermomechanical properties, which contribute to the successful fabrication of advanced nanocomposite materials [2,3]. Another type of thermoset polymer used to reinforce as well as modify a material’s performance is polyester. With its high thermal and mechanical properties, it is both simpler and cheaper to use than epoxy, and polyester can be used as the second matrix phase mixed with epoxy to create a general matrix structure of composite materials [4,5,6].

There are studies in which GNPs were used as fillers to enhance the properties of the epoxy matrix [7,8]. The new generation of hybrid composite materials with versatile mechanical, thermal and electrical properties are of particular interest to researchers [9,10].

The thermal conductivity of epoxy nanocomposites having 0.5 and 1 wt % of silica-coated MWCNTs was found to be enhanced by 51% and 67%, respectively, by Cui *et al.* [11]. Viswanath *et al.* [12] presented a thermomechanical and electrical study of epoxy/hyperbranched polyester. Rahman *et al.* [13] optimized the mechanical properties of epoxy reinforced with E-glass and amino-functionalized MWCNTs. The MWCNTs were aligned in the epoxy resin to achieve the required properties by Park *et al.* [14]; the thermal conductivity of epoxy/MWCNT nanocomposites at room temperature (RT) was observed to be 55 W/mK; the stretched MWCNT-epoxy sheet showed a value of 100 W/mK, whereas pure epoxy was 0.11 W/mK at RT.

In most previous studies, thermoset polymers did factor in the critical investigation of the performance of hybrid matrix materials made with epoxy and polyester. In this research, the added filler of GNPs was used to reinforce the tensile strength and thermal conductivity of the hybrid epoxy/polyester composite. The GNPs were mixed at different weight percentages with epoxy and polyester using high intensity ultrasonication. The resulting experimental specimens were obtained by means of the hot press casting method.

The primary interest of this paper was to characterize the effect of graphene nanoplatelets on the thermal and mechanical properties of the epoxy/polyester hybrid composite. Tensile tests were performed to evaluate the mechanical performance, and thermogravimetric analysis (TGA) and dynamic mechanical analysis (DMA) were carried out to evaluate the thermal properties. Additionally, the morphological characteristics of the surface structure of the nanocomposite samples in the tensile test were observed by a scanning electron microscope (SEM). The important contribution of this study is the successful fabrication of a hybrid matrix nanocomposite material of thermoset resins reinforced with the GNP filler. The nanocomposite specimens fabricated at a low cost evidenced high mechanical strength, good thermal conductivity and stability, making them suitable for applications in the manufacturing of components for electronic circuit boards and transportation.

## 2. Materials and Methods

### 2.1. Material Preparation

**Figure 1:**
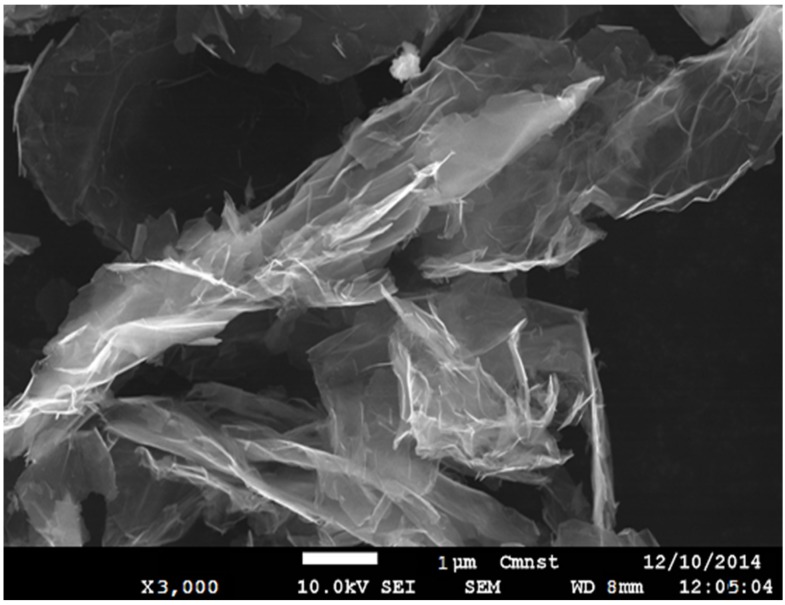
Scanning electron microscope image of pristine graphene nanoplatelets.

The graphene nanoplatelets (GNPs) used in this study were Grade 4 (Figure 1), purchased from Cheap Tubes Inc. (Cambridgeport, VT, USA), with an average plate diameter in the range of 1 to 2 µm and over 99% pure. The materials for creating the matrix structure of the composite consisted of epoxy 6620, hardener AH150 and unsaturated polyester resin (UPR) GC-0421, all purchased from Golden Innovation Business Co. (Taibei, Taiwan). As suggested by the manufacturer, some modification materials were used: an accelerator of cobalt naphthenate (6%) and a catalyst of methyl ethyl ketone peroxide (MEKP). To easily disperse the GNP filler in the resin, it was necessary to use methyl ethyl ketone (MEK) as a dissolution.

**Figure 2:**
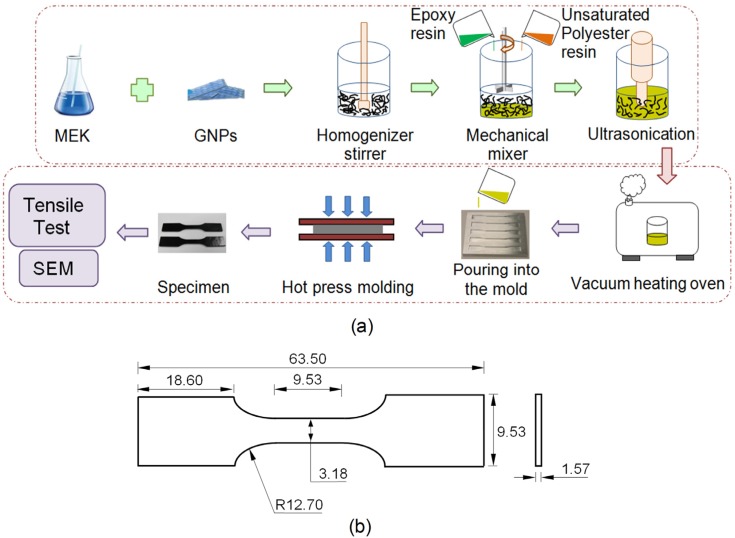
(**a**) Schematic showing the manufacturing process of the nanocomposite; (**b**) dimensions of specimen in ASTM standard Type V (mm).

**Figure 3:**
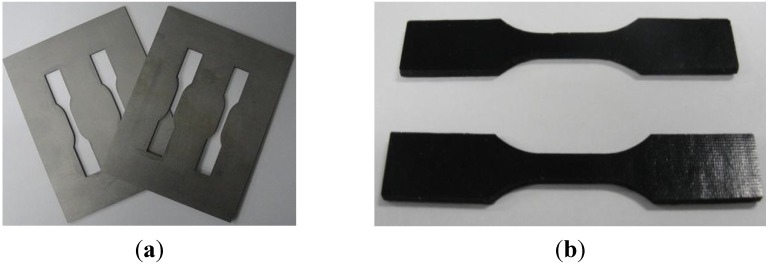
(**a**) Aluminum mold; (**b**) nanocomposite specimens.

Figure 2a shows the whole process of the experimental specimen preparation. The predetermined amounts of GNPs, epoxy and UPR were mixed together in a suitable beaker. The stipulated amounts of GNP and epoxy/polyester blend were mixed thoroughly with a magnetic stirrer for about 1 h at a temperature of 60 °C. The beaker was then placed in an ultrasonicator at a high intensity for 1½ h set at a pulse mode (9 s on/9 s off). An external cooling system was employed by submerging the beaker containing the mixture in an ice bath to avoid the temperature rising during the sonication process. When the process was finished, all agents, including the hardener, accelerator and catalyst, with a weight ratio of 2:1/1/1, were added to the previous epoxy/polyester mixture. An aluminum mold (Figure 2) of the required dimensions was used for the making of samples on par with the American Society for Testing and Materials (ASTM) standard. The mold was covered with a mold-releasing agent so the samples could be easily removed. The completed mixture was poured into the mold. The 2-part casting mold was maintained under a pressure of 0.8 MPa for 24 h at room temperature. To complete the curing process and eliminate residual stresses due to heat, the experimental samples (Figure 3b) were post cured at 90 °C for 3 h.

### 2.2. Mechanical Test

The experimental set up for the tensile test was done on an Instron 5566-CN2081 machine (Instron Company, Hsinchu, Taiwan) with a standard of ASTM D638 (Type V). The following parameters were set for the experiment: clamp length of 26.3 mm, tensile speed of 0.5 mm/min and room temperature of 30 °C. For each content of filler, the tensile test was carried out on at least five specimens; the result was the average value of the five measurements.

### 2.3. Thermal Analysis

The thermal characteristics of the GNP/epoxy/polyester-blended nanocomposites were studied using dynamic mechanical analysis (DMA) (TA Instruments Co., New Castle, PA, USA) and thermogravimetric analysis (TGA) (TA Instruments Co., New Castle, PA, USA).

DMA was performed using a TA Instruments 2980, operated in the three-point bending mode at a frequency of 1 Hz. The experimental data were obtained at room temperature to 160 °C at a scanning rate of 10 °C/min. The specimens for the bending test had the nominal dimensions of 2 mm × 15 mm × 6 mm. TGA was used to investigate the thermal decomposition behavior of the nanocomposite blend. Tests were done with a TA Instruments TGA2950 at a heat rate of 10 °C/min in a temperature range of 30 to 600 °C. A sample of 5 to 10 mg was used for each run. The weight change was recorded as a function of temperature.

## 3. Results and Discussion

### 3.1. Mechnical Properties

**Figure 4:**
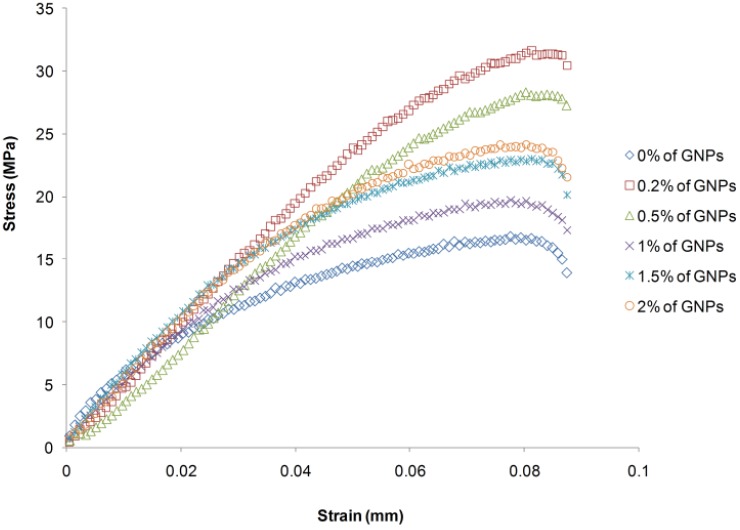
Stress-strain curves from tensile test.

The stress-strain responses of the nanocomposite specimens in the tensile tests are shown in Figure 4. The tensile properties of the nanocomposites at different weight percentages of filler were transformed in a similar way. Initially, the tensile stress increased in a linear style, showing an elastic deformation stage of the material. Then, the stress reached the maximum value and decreased rapidly to the complete failure state. The results of the tensile tests showed the nanocomposite specimens to have an elastoplasticity property and to be slightly brittle.

The analysis data of the 30 experiments, achieved using Nexygen Plus software with a high reliability of over 95%, are shown in Table 1.

**Table 1:** Dependence of the tensile strength on the filler ratio of graphene nanoplatelets (GNPs).

% of GNPs	Tensile strength (MPa)
0%	17.009
0.2%	31.775
0.5%	28.489
1%	19.914
1.5%	18.900
2%	18.492

The dependence of the tensile strength on the filler content of GNPs is shown in Table 1. When the weight percentage of GNPs was in the range of 0% to 0.2%, the tensile strength of the nanocomposite samples increased rapidly. When the content of GNPs increased to a range of 0.2% to 1%, the tensile strength decreased sharply. In a weight percentage range of 1% to 2%, the change in tensile strength was slight. The maximum value of tensile strength was achieved with a GNP content of only 0.2%. This value showed an increase of approximately 86.8% over the tensile strength of the specimens without added filler. The enhancement of the mechanical properties of the nanocomposites was due to the homogeneous dispersion of the reinforcement material and the good interaction between the reinforcement and the matrix, which was also confirmed by Gojny and Schulte [15].

### 3.2. Thermal Properties

**Figure 5:**
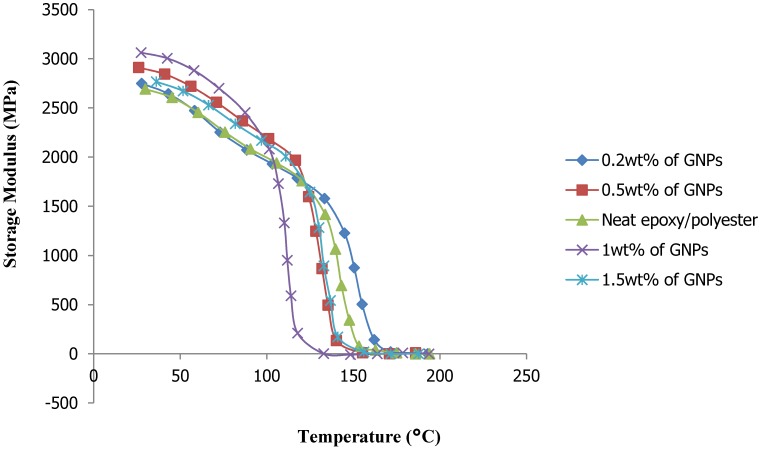
Storage modulus *vs.* temperature plots of GNP/epoxy/polyester nanocomposite.

Figure 5 presents the DMA results of the relationship between the storage modulus and temperature with different weight percentages of GNP filler. The plots show the storage modulus as a function of the filler content. With GNP filler increases from 0.2% to 1%, the storage modulus tended to increase. When the GNP content exceeded 1%, the storage modulus tended to decrease markedly. Here, epoxy/polyester nanocomposite reinforced with 1 wt % of GNP content achieved an increase of up to 3100 MPa of storage modulus at a temperature of 30 °C.

**Figure 6:**
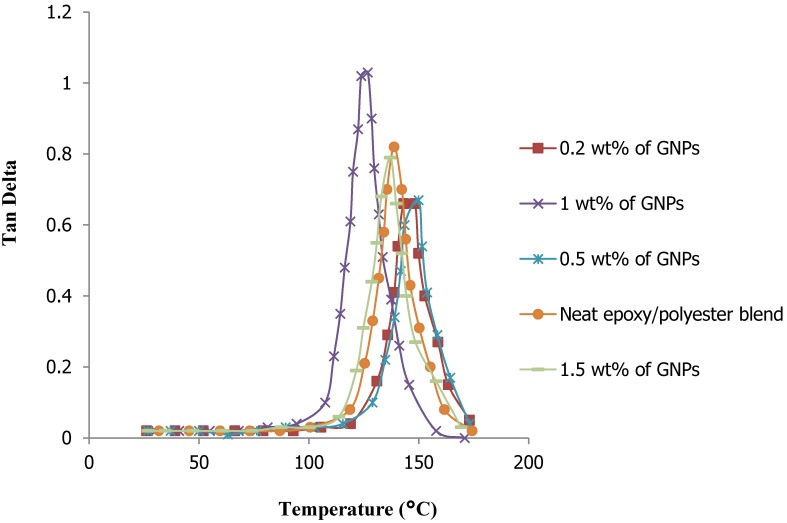
Relationship between the loss factor (tan*δ*) and temperature of GNP-reinforced hybrid composite specimens.

The loss factor curves (tan*δ*) of all the nanocomposites were obtained by DMA, as shown in Figure 6. The maximum value of the glass transition temperature (T_g_) was identified in the peak position of tan*δ*. This value was observed in the nanocomposite with filler content of 1.5%. As can be seen in Figure 6, the peak height of tan*δ* decreased with an increase in the GNP weight percentage, while the width of the loss factor showed little variation with the changes in nano-filler content. The peak factor, Γ, defined as the full width at half maximum of the tan*δ* peak divided by its height, can be qualitatively used to assess the homogeneity of the epoxy/polyester network. The low peak factor of the neat epoxy/polyester blend indicated that the crosslink density and homogeneity of the resin network were high.

**Figure 7:**
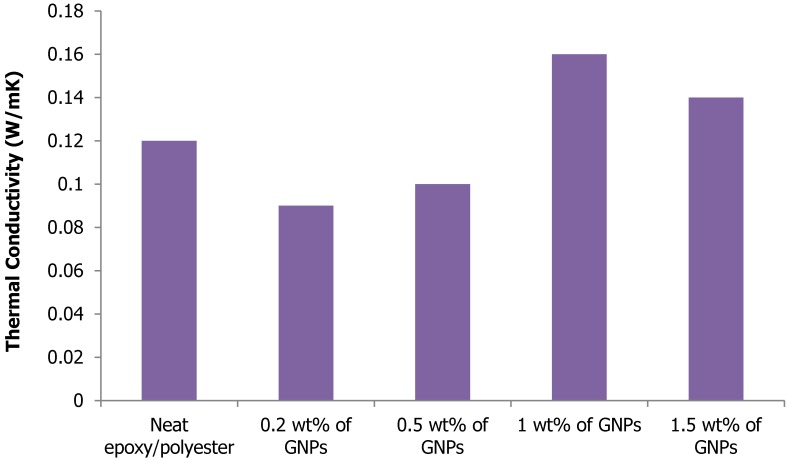
Thermal conductivity of nanocomposites.

Figure 7 shows the thermal conductivity of the epoxy and its nanocomposites prepared by different processing conditions. The thermal conductivity of the 0.2 wt % GNP sample and 0.5 wt % GNP was found to be 0.09 W/mK and 0.104 W/mK, respectively, lower than that of the neat epoxy/polyester (0.12 W/mK) due to the debonding between the reinforcement and the matrix. It was also observed that the thermal conductivity of the 1 wt % GNP sample increased with the reinforcement of GNPs, and the enhancement was significant as compared to that of the other samples. This enhancement of thermal conductivity was due to the homogeneous dispersion of GNPs in the epoxy/polyester resin and the bonding between them. The enhancement of thermal conductivity was found to be 33.3% for 1 wt % GNP sample compared to the neat epoxy/polyester.

**Figure 8:**
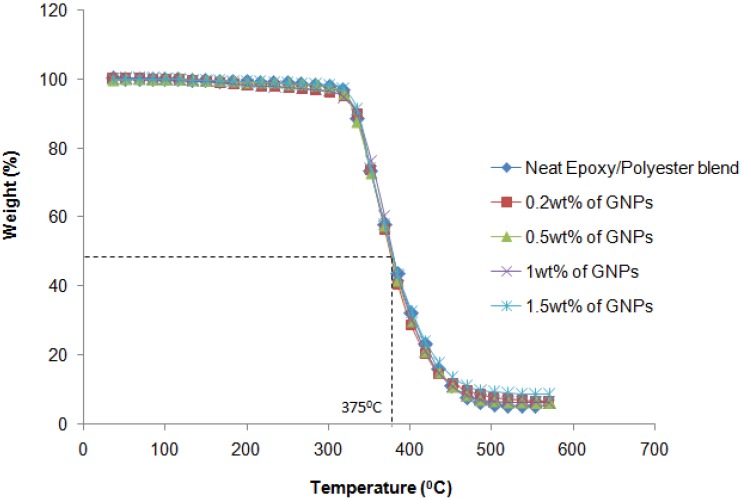
Thermal stability of nanocomposites.

Thermogravimetric analysis (TGA) was also performed to evaluate the thermal stability of all the fabricated nanocomposite samples, as shown in Figure 8. These samples were confirmed as having a 48% weight loss, evidence of the destruction in composite structure when affected by heat. Moreover, as this figure shows, the decomposition temperatures were almost the same for all the nanocomposites in the experiment, which proved that the heat destruction caused to the resulting nanocomposites in this study was not dependent on the content of fillers.

### 3.3. Morphological Characteristics

**Figure 9:**
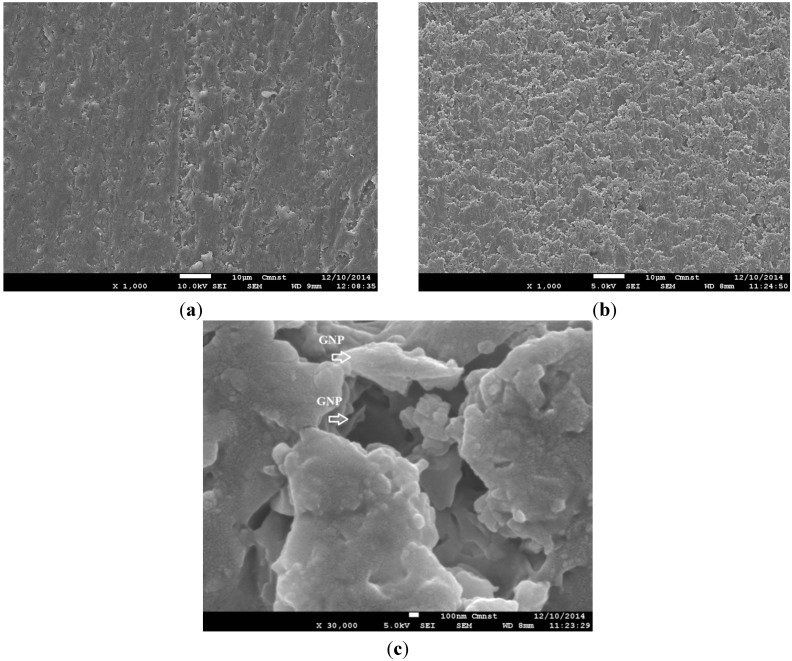
SEM graphs of (**a**) neat epoxy/polyester; (**b**) epoxy/polyester composite reinforced with 0.2% GNPs at a magnification of 1000; (**c**) epoxy/polyester composite reinforced with 0.2% GNPs at a magnification of 30,000.

Figure 9 shows the results of the morphological structure analysis of the fracture surface using SEM. The observed results were consistent with the obtained data of previous tensile tests. A relatively smooth surface can be seen in Figure 9a for the neat matrix material of epoxy and polyester mix without fillers, while in Figure 9b the rather rough fracture surface of the nanocomposites reinforced by 0.2% GNP filler is evident in the brittle fracture-style development of this material. The agglomeration of fillers negatively affected the mechanical properties of the nanocomposite. This phenomenon usually occurs when there is a large increase in the filler percentage. Figure 9c shows an SEM image of the 0.2% GNP-reinforced nanocomposite at a magnification up to 30,000. It can be seen from this image that the GNP filler was completely covered by the matrix phase, indicating a good adhesion between the two structural phases, which contributed to the enhancement of the mechanical properties of the produced nanocomposites.

## 4. Conclusions

This research used a nano-filler of GNPs to reinforce an epoxy/polyester blend. The tensile strength of the hybrid matrix nanocomposite reaches the maximum value with a very small amount of GNP filler added. When the weight percentage of the filler exceeded this determined value, the tensile strength of the nanocomposite decreased markedly and the material became brittle. The tensile strength of the GNP-reinforced epoxy/polyester blend nanocomposite reached approximately 86.8% with an addition of only 0.2% GNP content, as compared to neat epoxy/polyester. The DMA results indicated an improvement of up to 3100 MPa in the storage modulus for the epoxy/polyester nanocomposite reinforced with 1 wt % GNPs. The TGA results confirmed the thermal stability of the resulting nanocomposite specimens, regardless of the weight percentage of the GNPs. Additionally, as observed in the SEM images, there was good adhesion between the fillers and the resin. Therefore, the novel materials created in this study were of high mechanical strength, good thermal conductivity and stability, and can be used to produce high-performance components which are light weight, high quality and low cost for applications in electronic circuit boards and transportation.
